# Lessons on Non-Progression of HIV Disease from Monkeys

**DOI:** 10.3389/fimmu.2013.00064

**Published:** 2013-03-13

**Authors:** Pramod N. Nehete, Shailbala Singh, K. Jagannatha Sastry

**Affiliations:** ^1^Department of Veterinary Sciences, The University of Texas MD Anderson Cancer CenterBastrop, TX, USA; ^2^Department of Immunology, The University of Texas MD Anderson Cancer CenterHouston, TX, USA

Rhesus macaques infected with simian immunodeficiency virus (SIV) represent the most widely used model for studies related to understanding infection, pathology, immunology, and intervention strategies for HIV infection and AIDS in humans. This model recapitulates the significant impact of host immunogenetics and inter-individual variations on the natural disease course of HIV-AIDS in humans. In addition to the parallel kinetics for fast- and slow-progressing disease course observed in majority of animals, a small subset of animals exhibit long-term non-progression (LTNP) or elite control (EC) status defined as low to undetectable viral loads in the absence of any interventions for prolonged periods. Therefore, the rhesus macaque model has been and continues to be the most extensively adopted experimental system for studies to not only confirm observations from HIV-ADIS in humans but also to validate several mechanistic features of the infection and disease course, which otherwise would be impractical and immoral to be performed in humans.

The natural EC of HIV-1 in humans and of SIV in rhesus macaques has been attributed to the contributions of variety of host and viral factors (Betts et al., 2006; Saksena et al., 2007; Freel et al., 2010). Virus-specific CD4^+^ T helper cells are central for maintaining effective immunity in case of a number of chronic viral infections including HIV, where infected individuals that control viremia in the absence of antiviral therapy exhibit persistent and vigorous HIV-specific CD4^+^ T cell responses, in particular to the viral core protein p24 that display polyfunctionality in terms of secreting multiple cytokines and chemokines (Kalams and Walker, 1998). Employing the SIV-rhesus model, Louis Picker's group showed that strong and early containment in terms of undetectable plasma viral loads as well as prolonged protection against pathogenic SIV challenge in monkeys immunized with SIV DNA delivered by employing rhesus CMV and Adenoviral vectors correlated with high frequencies of virus-specific effector memory CD4^+^ T cell responses within the potential sites of SIV replication (Hansen et al., 2011). In our lab, studies testing peptide-based vaccine (Nehete et al., 1998, 2001, 2005, 2008) and adenovirus vectored vaccines (Mercier et al., 2007; Weaver et al., 2009) against systemic or mucosal challenge with different strains of simian human immunodeficiency viruses (SHIV ku2, SHIV89p, and SHIV 162p3), revealed strong correlation for virus control, to undetectable levels in significant population of animals, preferentially with antigen specific cellular immune responses (CD4^+^ and CD8^+^ T cells). We also assessed functional characteristics of memory T cells subsets as potential prognostic markers for changing viral loads and/or disease progression using the SHIV-infected rhesus macaque model and observed that functional impairment of CD4^+^ T cells in general, and of central memory subset in particular, may be a potential indicator/predictor of chronic infection with immune dysfunction, which could be assayed relatively easily using non-specific PMA + I stimulation (He et al., 2011).

Two other immune cell types recognized for potential contributions to influence virus control in SIV infected rhesus macaques and HIV-infected humans include CD4^+^ T cells subsets that either produce IL-17, Th17 cells, or exert immunosuppressive function, CD25^+^FoxP3^+^ regulatory T cells or T-reg (Cecchinato and Franchini, 2010). Depletion of Th17 cells in humans as well as macaques, relatively soon after virus exposure, has been associated with the dissemination of microbial products from the infected gut, thereby adding to the systemic immune activation and disease progression. On the other hand, T-regs have been associated with compromised antiviral T cell responses. Substantive studies in the non-human primates combined with epidemiological observations in humans suggest that both these cells subsets, through influencing innate and adaptive immune responses, significantly impact the inflammatory milieu of the host to ultimately affect the outcome of the immunodeficiency virus infection (Favre et al., 2009; Dandekar et al., 2010; Kanwar et al., 2010).

Clinically healthy HIV-infected individuals that maintain stable high CD4^+^ T cell counts also exhibit strong and early onset of anti-HIV CD8^+^ T cell responses pointing to their protective role, which prompted elegant experiments that tested *in vivo* CD8^+^ T cell depletion in SIV infected rhesus macaques demonstrating the critical role of these immune cells in the early control of infection (Deeks and Walker, 2007; Freel et al., 2010). While the role of CD4^+^ T cells in cell-mediated immunity is manifested in terms of production of a variety of cytokines, CD8^+^ T cells are more frequently recognized for their cytolytic property even though they are capable of multiple functions that include production of a variety of cytokines and soluble factors to restrict viral replication in infected cells (Buchbinder et al., 2008). In this regard, Watkins group (Goulder and Watkins, 2004) using *in vitro* assays based on cytokine production by the CD8^+^ T cells demonstrated suppression of viral replication and also showed that CD8^+^ T cell clones, from monkeys in the elite SIV control group, recognizing the same epitope differ significantly in their capacity to suppress SIV replication. These studies further demonstrated that the *in vitro* efficacy of the CD8^+^ T cells to suppress virus replication is not necessarily tied always to production of cytokine most commonly assayed, such as IFN-γ, TNG-α, or IL-2 (Goulder and Watkins, 2004).

The unique strength of the virus-specific CD8^+^ T cell immunity for virus control in LTNP and EC has been most widely elaborated in the literature to be associated with host genetic factors, specifically expression of certain class I major histocompatibility complex (MHC-1) alleles (Migueles et al., 2000; Fellay et al., 2009). It has been well recognized that individuals expressing *HLA-B27*- and -*B57* alleles are among the elite controllers of HIV replication, implying that CD8^+^ T cell responses restricted by these host genes exert potent anti-HIV effects. Analogous to these observations are studies in the SIV-rhesus model demonstrating that animals expressing Mamu-A*001 and Mamu-B*08 exhibit low to undetectable viral loads without experimental interventions, so much so that 50% of Mamu-B*08-positive rhesus macaques of Indian origin were shown to effectively control SIVmac239 replication to become EC (Loffredo et al., 2008). Such parallels between human and rhesus MHC alleles contributing for virus control, defined as significantly lower set-point viral load and prolonged survival time, were noted specifically for HLA-B*14, and -B*57 in humans and Mamu-A*001, A*002, A*008, A*011; B*001, B*003, B*004, B*008, B*017, B*029, DRB*w201, DRB1*0401/06/11, and DPB1*06 in macaques (Goulder and Watkins, 2008). The most compelling evidence, beyond the genetic linkage studies, for the contribution of host immune responses restricted by specific MHC alleles is presented in recent studies from the Watkin's group (Mudd et al., 2012). In this study, Mamu-B*08 expressing rhesus macaques when vaccinated with three peptide sequences restricted by Mamu-B*08 generated high frequencies of corresponding CD8^+^ T cell response in blood, lymph nodes, and colon that was associated with efficient viral control. Furthermore, in two of the eight animals from the study viral rebounds during the chronic infection phase correlated with the emergence of escape variants mutated specifically in all three epitope sequences, demonstrating a direct role for CD8^+^ T cells in viral control.

The most powerful recognition for the strength of the SIV-rhesus model for HIV-AIDS research comes from studies by the Sette group (Sette et al., 2005) that analyzed a panel of ∼900 peptides to show a high degree of sequence homology for the Mamu-B*08-restricted epitopes from SIV to match the HIV peptide-binding motifs for HLA-B*2705 in humans, despite substantial sequence differences between these MHC alleles. Further detailed studies of the Mamu-B*08 peptide-binding motif enabled this group to identify six additional novel Mamu-B*08-restricted SIV-specific CD8^+^T cell immune responses directed against epitopes in Gag, Vpr, and Env. This remarkable similarity of peptide-binding motifs of MHC alleles in two different species highlight not only the important role for the specific peptides and related immune responses, but also the relevance of macaques expressing such unique MHC alleles as model to examine EC of immunodeficiency virus replication.

One of the HIV encoded genes, nef, has long been recognized to be important for viral infectivity, though it was shown to be not required for *in vitro* viral replication in cell cultures. Clinical epidemiological studies identified HIV-infected asymptomatic individuals to harbor virus with mutated nef gene sequences, specifically point mutations involving the cysteine residue at position 138 (Cys-138), over a sustained period of 3 years of infection without any disease progression (Premkumar et al., 1996). Incidentally, this Cys-138 mutation in the nef gene was also identified in the chimpanzee immunodeficiency virus (CIV), another member of the lentivirus family with close similarity to HIV, but chimpanzees infected with this virus do not progress to AIDS (Premkumar et al., 1996). Furthermore, SIVagm, an SIV variant that commonly inhabits African Green monkeys expresses nef gene with multiple cysteine residues including the Cys-138 mutation, does not cause AIDS in this monkey (Premkumar et al., 1996). These observations were confirmed by experimental evidence from studies employing the SIV-rhesus model, notably by the Desrosier's group (Kestier et al., 1991) who showed that SIV variants with nef gene deletions, but not wild type SIV, failed to maintain high viral loads necessary to cause AIDS in infected adult rhesus monkeys (Kestier et al., 1991). Based on these reports, several studies explored the attenuated SIV strains with deletion of the nef gene alone or along with additional viral genes as potential candidate vaccines.

The power of non-human primates as the experimental animal models of choice for HIV-AIDS, specifically to understand the non-progression of infection/disease, extends beyond the rhesus macaques. The sooty mangabeys and African green monkeys are two non-human primate species that are the natural hosts for SIV infection where the virus exhibits high replication capacity with transient or severe CD4^+^ T cells loss but without clinical signs of simian AIDS. Valuable information has been gathered from studies in these models for SIV infection and the potential of host and viral factors to serve as correlates of protection and/or EC. In sooty mangabeys CD3^+^CD4^−^CD8^−^ T cells (double-negative T cells) capable of producing Th1, Th2, and Th17 cytokines seem to partially compensate for the loss of CD4^+^ T cell function in maintaining disease-free status exemplified by controlled viral replication and low levels of immune activation (Milush et al., 2011). These studies bring to focus the importance of double-negative T cells and studies to better understand their modulation in HIV-AIDS (Silvestri et al., 2005; Barry et al., 2007). Similarly, studies in African green monkeys emphasize the lack of chronic T cell activation as a critical determinant for the difference between the non-pathogenic SIV in this model and the pathogenic HIV-1/SIV infections in humans and rhesus macaques. Accordingly, this is an active area of research pursued by several groups focusing on the innate immune system and the induction of immunosuppressive mediators, specifically during early SIV infection (Kornfeld et al., 2005; Jacquelin et al., 2009).

## Conclusion

Non-progression to disease in HIV-infected individuals is a rare but highly important observation for designing informed and logical strategies for controlling and even preventing HIV-AIDS. Studies in the non-human primate models thus far revealed parallels for all stages of HIV disease progression in humans including that rare non-progression. Important lessons learned from these models included identification, with certainty, of contributing host immunological and genetic factors in more practical terms. Most significant among these are the demonstration of the relevance of CD8^+^ T cells in controlling infection and the practical implications of protective MHC alleles, the later through studies demonstrating the protective efficacy of vaccine studies employing relevant SIV antigens. Similarly, studies in the natural hosts of SIV (sooty mangabeys and African green monkeys) that despite indistinguishable susceptibility to infection from experimental hosts (rhesus macaques), revealing the importance of specific immune cells subsets and chronic immune activation for LTNP. More controlled experimentation, only possible with these non-human primate animal models but not in human trials, will further help in depth understanding of these factors for designing strategies to manipulate them toward inducing desirable protective responses applicable to HIV-AIDS in humans.
